# Multidisciplinary evaluation of *Clostridium butyricum* clonality isolated from preterm neonates with necrotizing enterocolitis in South France between 2009 and 2017

**DOI:** 10.1038/s41598-019-38773-7

**Published:** 2019-02-14

**Authors:** Michel Hosny, Jacques Yaacoub Bou Khalil, Aurelia Caputo, Rita Abou Abdallah, Anthony Levasseur, Philippe Colson, Nadim Cassir, Bernard La Scola

**Affiliations:** 1 Aix-Marseille Université UM63, Institut de Recherche pour le Développement IRD 198, Assistance Publique – Hôpitaux de Marseille (AP-HM), Microbes, Evolution, Phylogeny and Infection (MEΦI), Institut Hospitalo-Universitaire (IHU) - Méditerranée Infection, Marseille, France; 2 Aix-Marseille Université UM63, Institut de Recherche pour le Développement IRD 198, Assistance Publique – Hôpitaux de Marseille (AP-HM), Vecteurs – Infections TROpicales et MÉditerrannéennes (VITROME), Service de Santé des Armées, Institut Hospitalo-Universitaire (IHU) - Méditerranée Infection, Marseille, France

## Abstract

The association between *Clostridium* species identification from stool samples in preterm neonates and the occurrence of necrotizing enterocolitis has been increasingly reported. To confirm the specific impact of *Clostridium butyricum* in this pathology, selective culture procedure was used for Clostridia isolation. Whole-genome analysis was employed to investigate genomic relationships between isolates. Stool samples from present study, as well as from previously investigated cases, were implicated including 88 from preterm neonates with necrotizing enterocolitis and 71 from matched controls. Quantitative real-time polymerase chain reaction was performed to evaluate the presence of *C*. *butyricum* from stools of new cases. *Clostridium* species prevalence isolated by culture was compared between patients with necrotizing enterocolitis and controls. By combining results of both culture and quantitative polymerase chain reaction methods, *C*. *butyricum* was significantly more frequent in stool samples from preterm neonates with necrotizing enterocolitis than in controls. Whole-genome analysis of 81 genomes including 58 neonates’ isolates revealed that cases were clustered depending on geographical origin of isolation. Controls isolates presented genomic relations with that of patients suggesting a mechanism of asymptomatic carriage. Overall, this suggests an epidemiology comparable to that observed in *Clostridium difficile* colitis in adults.

## Introduction

Necrotizing enterocolitis (NEC) is a multifactorial intestinal disease that occurs in 4–12% of preterm neonates, with a high attributable mortality especially in patients with very low birth weight (*i*.*e*. 500–1000 g)^1,2^. Recent studies propounded the idea of bacteriological etiology, mostly microbiota dysbiosis during NEC episodes, leading to the generation of inflammatory process^3,4^. In recent years, several bacterial species have been suspected of being associated with NEC outbreaks, especially Clostridia like *Clostridium butyricum*, *Clostridium perfringens* and *Clostridium neonatale*^4,5^. Zhao-Fleming *et al*. also reported a significantly higher frequency of strictly anaerobic species by using 16S rRNA sequencing to identify bacteria from patients with necrotizing infections^6^. Cassir *et al*. described a relationship between the presence of *C*. *butyricum* in stool samples from preterm neonates and the occurrence of NEC, using a combination of three techniques (16S rRNA pyrosequencing, culturomics and qPCR). However, neither the pathogenicity nor the epidemiology of this relationship have yet been totally resolved^7^. In addition to Clostridia, Gram-negative bacterial species were also identified especially, *K*. *pneumoniae*, *E*. *cloacae* and Uropathogenic *Escherichia coli*^8,9^.

In present work, we conducted a multidisciplinary study on a cohort of NEC and control preterm neonates stool samples that we investigated for *C*. *butyricum* in stool by qPCR, a small fraction of samples only being previously tested by culture^7^. The main objective of the current investigation on this cohort was to isolate the qPCR-detected *C*. *butyricum* in order to perform strain typing by whole genome sequencing on isolates and to correlate it with the date of sampling as the geographical origin of the isolates. Indeed, in a preliminary work done on 16 strains, we suspected isolates to have a clonal origin isolates^10^. For this, we used a heat-shock selective culture protocol that allowed us also to isolate *C*. *butyricum* but also some other Clostridia previously also suspected to be associated to NEC. We took the opportunity in the present work to add additional cases received between 2015 and 2017. Then, the association between *C*. *butyricum* and the occurrence of NEC was reassessed and typing data were analysed together.

## Results

### Evaluation of *Clostridium* species community in samples from preterm neonates

Combination of culture and qPCR results allowed the identification of a strong association between the presence of *C*. *butyricum* in stool samples from preterm neonates and the occurrence of NEC, when compared to controls (66/88; 75% *vs*. 8/71; 11%; *p* < 0.001 respectively) (Table 1). Overall, 58 strains of *C*. *butyricum* were isolated including 52 (59.1%; n = 88) from NEC patients and 6 (8.45%; n = 71) from controls (*p* < 0.001) (Table 2). By comparing means of cycle thresholds (Ct), the density of *C*. *butyricum* was lower in controls stool samples than in NEC (32.938 and 29.182 respectively, *p* value = 0.0344). The frequency of *C*. *butyricum* in this later seems to be associated with the administration of antibiotics (*p* value = 0.027), which is not the case in controls (*p* value = 0.838) (Supplementary Table S1). As for the other previously proposed NEC-associated *Clostridium* species, we isolated four strains (4.5%) of *C*. *neonatale* from NEC patients and one (1.14%) from controls (*p* = 0.26). *Clostridium perfringens* was detected equally in patients with NEC and controls (respectively 8; 9% and 6; 8.4%). This was also the case for *Clostridium paraputrificum* (1; 1.1% and 1; 1.4% respectively). Finally, we identified other Clostridia from the stools of patients with NEC: *Clostridium sardiniense* (1; 1.1%), *Clostridium tertium* (1; 1.1%) and *Clostridium difficile* (2; 2.3%). In particular, 7/8 and 1/6 of *C*. *perfringens* from the NEC patients and controls, respectively, were co-isolated with *C*. *butyricum* in the same sample.

**Table 1:** Detection of *Clostridium butyricum* by qPCR and Heat-shock.

	*C. butyricum qPCR*		*C. butyricum culture*		*C. butyricum* qPCR + culture	
	NEC (n = 88)	Controls (n = 71)	NEC (n = 88)	Controls (n = 71)	NEC (n = 88)	Controls (n = 71)
Positive	63 (71.6%)	8 (11.3%)	52 (59.1%)	6 (8.4%)	66 (75%)	8 (11%)
Negative	25 (28.4%)	63 (88.7%)	36 (40.9%)	65 (91.6%)	22 (25%)	63 (89%)
P value	p value < 0.001		p value < 0.001		p value < 0.001	

NEC: necrotizing enterocolitis, qPCR: quantitative real-time polymerase chain reaction.

**Table 2:** Clinical Characteristics of necrotizing enterocolitis and controls preterm neonates.

Characteristics	NEC (n = 88) No. (%)	Controls (n = 71) No. (%)	*p* value
Gestational age +/− SD (weeks)	28.36 (+/−2.82)	28.24 (+/−2.93)	0.7864
Days of life +/− SD (days)	25.99 (+/−13.16)	23.99 (+/−11.61)	0.3166
Birth weight mean +/− SD (g)	1179 (+/−380)	1174 (+/−432)	0.950
Male sex	49 (55.7)	43 (60.5)	0.594
Very low birth weight (≤1500 g)	69 (78.4)	58 (81.7)	0.617
Pasteurized breast milk	59 (67)	49 (69)	0.372
Formula fed	9 (10)	13 (18)	0.139
Vaginal delivery	32 (36)	37 (52)	0.023
Antibiotics before collection	64 (73)	40 (56)	0.092

NEC: necrotizing enterocolitis.

### Core-genome and core-genome single-nucleotide polymorphism phylogenetic analysis

**Figure 1:**
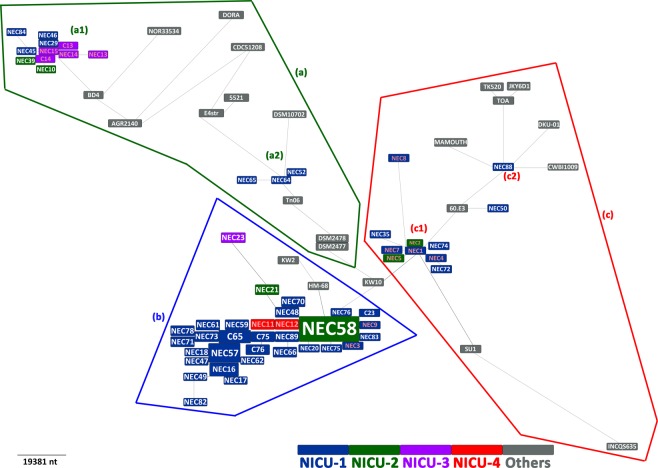
*Clostridium butyricum* geographic relationship based on core-genome single-nucleotide polymorphism phylogenetic analysis. The color of strain names represents the geographic zone of isolation. (Marseille: NICU-1, NICU-2; Nice: NICU-3; Montpellier: NICU-4). Red strain names represents the 16 isolates sequenced Cassir *et al*.^7^. NEC: necrotizing enterocolitis, NICU: neonatal intensive care units.

**Figure 2:**
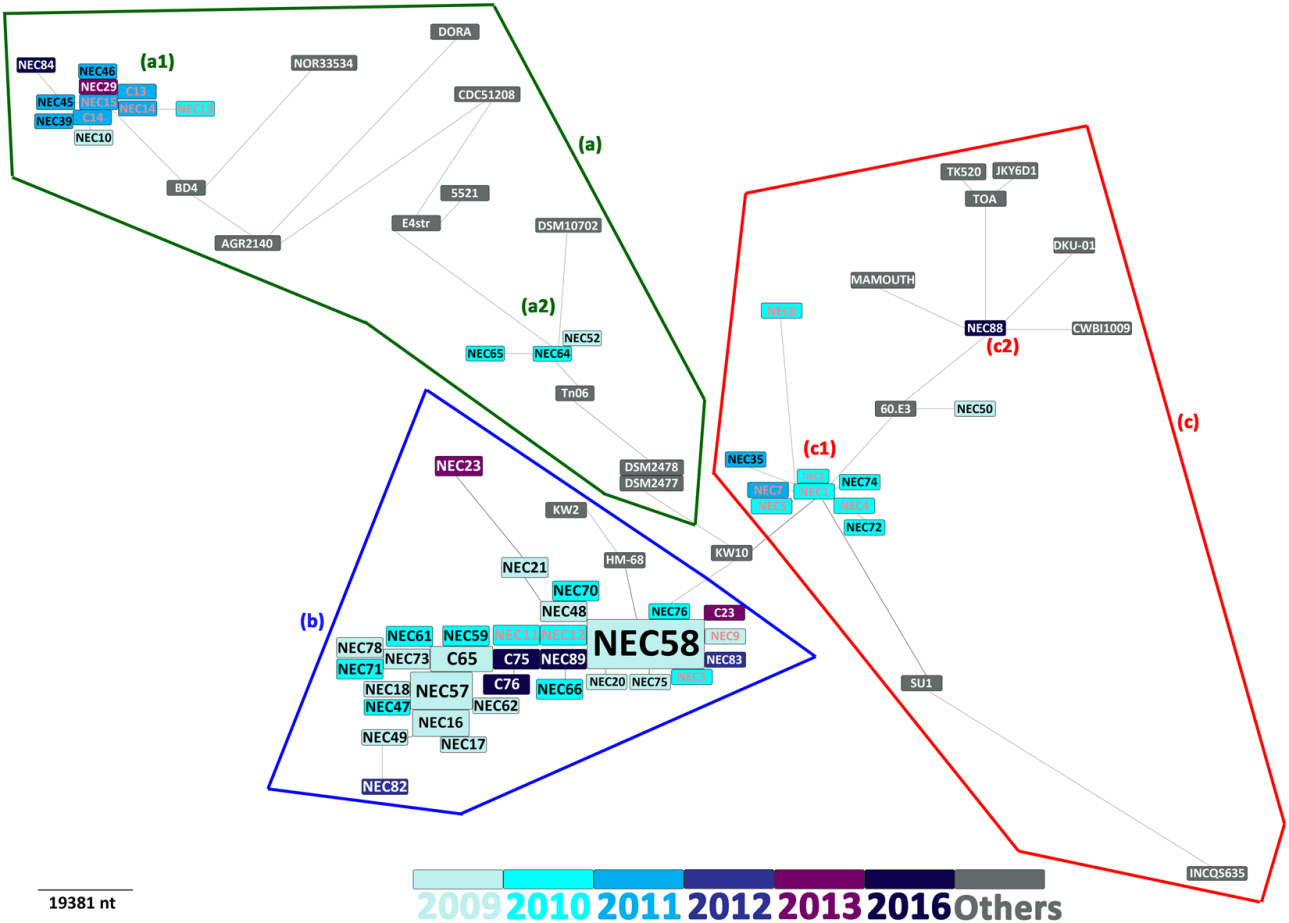
*Clostridium butyricum* temporal relationship based on core-genome single-nucleotide polymorphism phylogenetic analysis. The color of strain names represents the timing of isolation. Red strain names represents the 16 isolates sequenced Cassir *et al*.^7^. NEC: necrotizing enterocolitis, C: control.

The average length of genomes included in this study was 4,676,116 bp. The greatest genome size was 5,214,902 bp (strain NEC23), the smallest genome size was 4,014,159 bp (strain DORA). An average of 4,363 open reading frames was predicted (Supplementary Table S2). Although strains isolated from Marseille (NICU-1 and NICU-2) were over-represented in this study, the eBURST analysis of core-SNP enabled the identification of three main *C*. *butyricum* clonal groups (a, b, c): group (b) included one cluster; cluster (b), thus both group (a) and (c) included two clusters; respectively cluster (a1, a2) and cluster (c1, c2) (Figs 1, 2 and Supplementary Figure S1–S3). The majority of NEC-associated *C*. *butyricum* (28/52, 54%) were clustered within the group (b). Genomic relationships were identified between NEC and control isolates, also grouped depending on geographic source of isolation. Strains isolated from the same NICUs were close to each other *i*.*e*. cluster (a1), (a2), (b), (c1) isolates detected from NICU-1 and 2 in Marseille, also, *C*. *butyricum* strains from NICU-3 and NICU-4 on the other hand in cluster (a1) and (b) (Fig. 1). Core-SNP temporal clustering revealed that highly-related strains were identified between 2009 and 2010 in group (b) (73.5%; 25/34), also, during 2010 in cluster (c1) (Fig. 2). Nearly all strains were grouped in clusters revealing their clonality whereas strains from other origin were dispersed and far from each another. The comparative analysis allowed the identification of hemolysin-encoding genes (hemolysin A, B, C and beta) in NEC and control isolates from all clusters.

### Genotyping using multispacer sequence typing phylogeny

MST phylogeny identified four sequence types (ST) where the strains had the same MST: NEC1, NEC3, NEC10 and NEC17 (Supplementary Figure S4 and S5). We compared the eBURST analysis of MST with that of core-SNP. Isolates were clustered into three groups centered on the Kwashiorkor strain (KW10): Group a’ included the same cluster as Group a, except for NEC1, NEC8, NEC35, NEC72 and NEC74; Group b’ included the same strains as Group b except for NEC29, NEC52, NEC64, NEC65 from Group (a) and NEC88 from Group (c); finally, Group c’ included NEC50.

## Discussion

Inclusion of additional cases and the use of a selective culture procedure for *Clostridium* isolation confirmed our previous results showing a significant association between *C*. *butyricum* and NEC in preterm neonates^7,10^. Indeed, 75% of NEC were positive using qPCR and culture, while this rate was only 11% in controls (*p* < 0.001). Culture technique emphasizes the specificity of qPCR, as 3.4% of NEC-associated *C*. *butyricum* were negative by qPCR and positive by culture. Reciprocally, qPCR validated the culture by detection of *C*. *butyricum* in 75.75% (50/66) from all positive samples. The advantage of this selective culture protocol allowed easy isolation of *C*. *butyricum* during NEC outbreaks as compared to culturomics technique which is highly fastidious^7^. The drawback of included culture method was the possibility to skip isolation of *in vivo* non-spore-form of Clostridia species. As well, the density of *C*. *butyricum* was higher in NEC than in controls (*p* value = 0.0344). The association between *C*. *butyricum*-linked NEC with antibiotics administration (*p* value = 0.027), suggested a mechanism of pathogenicity comparable to that of *C*. *difficile*, where cases of pseudomembranous colitis were significantly associated with previous antibiotic administration, especially lincosamides, third generation cephalosporins and fluoroquinolones. It would be interesting, in future studies, to evaluate the influence of antibiotics families in the occurrence of NEC. Additionally, culture enabled the isolation of other *Clostridia* (n = 25) previously suggested in linked with NEC that were not evaluated by Cassir *et al*.^7^. Among these, *C*. *neonatale* and *C*. *perfringens* were specifically suspected to be associated with NEC^4,5,11–15^. These species were infrequently isolated, the most predominant being *C*. *perfringens*, with a total of 14 isolates observed equally in both NEC and control groups. Moreover, it was co-isolated with *C*. *butyricum* in 62.5% of patients. This result differs from that of Sim *et al*., where the authors used a metagenomic approach to detect *C*. *perfringens* type A (3/12; 25%) from NEC samples^16^. It has been demonstrated that early colonization by *C*. *perfringens* affected the onset of NEC^14^. Furthermore, unbalanced gut microbiota in Lebanese neonates suffering from NEC have been reported, including *C*. *perfringens* widely identified in patients, despite the limited number of samples tested in this study^17^. *C*. *neonatale* was less prevalent than *C*. *butyricum* and *C*. *perfringens* and not significantly associated with NEC (NEC and controls, n = 4, 4.5% versus n = 1, 1.4% respectively, *p* = 0.5). However, it is difficult to draw conclusion from a limited number of isolates. *C*. *neonatale* was first identified by Alfa *et al*., during a NEC outbreak in a Canadian NICU. However, no control neonates were tested, and its pathogenicity was not fully investigated^15^, only its microbiologic features were studied^18^, particularly through comparative phenotypic features with *C*. *butyricum*^19^.

Genomic relations were previously reported by Benamar *et al*. on a limited number of strains (n = 32) including 16 isolated from NEC and controls^10^. Three clonal clades were identified. In the present work, 42 additional preterm neonates’ strains isolated from different geographic origin and time of isolation were analysed. Core-genome, core-SNP and MST phylogenetic analysis on an exceptional collection of *C*. *butyricum* strains (n = 81) confirmed genomic relationship between NEC-associated and controls isolates as the discrimination of the prior identified clades. Therefore, three main epidemic clonal groups were identified, including a large clonal cluster occurring between 2009 and 2010 (cluster (b)). This last clustered the majority of NEC-associated *C*. *butyricum* (28/52, 54%) identified as clade C in Benamar *et al*.^10^. In some cases, especially during September/October 2010, NEC occurred as an outbreak (n = 10) with highly related isolates. Other small epidemics clusters were detected with a limited number of strains (Groups a1, a2, c1 and c2). Additionally, strains isolated from the same NICU were highly related suggesting the existence of geographic-associated *C*. *butyricum* clonal lineages and epidemic transmission. This was confirmed by similarities observed between controls and NEC-associated isolates and highlighted a *C*. *butyricum* asymptomatic carriage of these genetically-related strains. Clusters (a2) and (c2) were not observed in Benamar *et al*.^10^ and corresponded to strains isolated after 2015. This finding confirms the temporal clustering.

The first NEC-associated *C*. *butyricum* outbreak was reported by Howard *et al*. in 1977, based on clinical examination and microbiologic analysis, since no genotyping methods were employed^20^. Gorham *et al*. reported 152 cases of *C*. *butyricum* hand-carriage isolated from 152 healthcare workers during a NEC outbreak^21^. Several studies showed that *C*. *butyricum* is a beneficial microorganism, especially MIYAIRI 588 strain, that prevents entero-hemorrhagic *E*. *coli*, *C*. *difficile* infections and gastric ulcers^22^. Conversely, other strains might contribute to the pathogenesis of several infectious diseases, such as botulism and NEC^22,23^. Recently, Wydau-Dematteis *et al*. reported the importance of the *dlt* operon of *C*. *butyricum* associated NEC by establishing the resistance of *C*. *butyricum* to antimicrobial peptides, lysozymes and vancomycin^24^. A high dose of butyric acid has also been demonstrated to have a cytotoxic effect on diverse cell lines, notably Caco-2 cells monolayers^25^. Genomic identification in *C*. *butyricum* of a toxin comparable to β-hemolysin secreted by *Brachyspira hyodysenteriae*^7^ suggests a similar possible mechanism for cytotoxicity^26^. Moreover, we previously reported a cytotoxic activity of *C*. *butyricum* supernatant tested by flow cytometry on Jurkat cells^7^. Such a toxigenic mechanism of Clostridia in NEC disease is supported by the findings of Heida *et al*., who observed a low density of Clostridia by fluorescent *in situ* hybridization^27^. Other toxins-related NEC cases have been suggested in association with different bacterial species such as toxigenic *E*. *coli*, *Pseudomonas aeruginosa*, *Serratia* spp., delta toxin-producing strains of coagulase-negative Staphylococci, *S*. *aureus* and pore-forming type A toxin *Clostridium perfringens*^28,29^.

The picture observed in *C*. *butyricum*-associated NEC resembles that of *C*. *difficile-* associated pseudomembranous colitis^30^. Similarly to *C*. *butyricum*-associated NEC, *C*. *difficile* hospital-associated isolates were found to be highly related with asymptomatic carriage, supporting the idea of a plausible transmission amongst hospitals as from environmental reservoirs^31,32^. This work is the first to note that the WGS was used to investigate probable *C*. *butyricum*-associated NEC outbreak, during a period of 8 years using SNP analysis. WGS is a revolutionizing large-scale genomic-based technique, highlighting the clonal transmission between reservoirs and patients^33^, as the identification of virulence factors^34^. In addition, we observed that MST techniques represent an alternative method as clusters were approximately validated by core-SNP analysis, but due to continuous reduction of costs, WGS will be probably preferred for routine use in future diagnosis.

## Conclusion

The key findings of this study were first, the confirmation of significant association between *C*. *butyricum* strains and the occurrence of NEC, then the genetic correlation between NEC and non-NEC-associated strains of *C*. *butyricum*. This suggested that cases emerge from a small number of genetically heterogeneous sources, validated the possibility of asymptomatic carriage and confirmed that most strains are hospital-acquired. These findings and significant association with the administration of antibiotics in NEC patients resembles epidemiology of *C*. *difficile* in adults.

## Methods

### Study design and patients

One hundred and fifty-nine preterm neonates with NEC and matched controls were enrolled, including neonates from the study of Cassir *et al*. (NEC n = 82, control n = 67)^7^, and 10 (NEC n = 6, control n = 4) received between 2015 and 2017 (Supplementary Table S3). The stool samples collected from 88 NEC and 71 controls were from four south-eastern regions of France (Marseille, Nice, Nîmes and Montpellier), including five neonatal intensive care units (NICUs) (Table 2, Supplementary Table S2 and Figure S6). Cases were defined as patients with suspected, definite or advanced NEC corresponding to Bell stages I, II or III. When feasible, stool samples were collected on the day of symptoms onset and stored at −80 °C. Controls were matched with patients by sex, gestational age, birth weight, days of life, feeding strategies, mode of delivery, and previous antibiotic therapy and NICU. All patients with NEC and controls were preterm neonates (under 37 weeks completed weeks of gestation). Since the dates of antibiotic administration were not recorded, patients were not distinguished for the duration of antibiotic therapy. None of the selected patients had received probiotics or been included in therapeutic protocols prior to stool collection and all were negative for routine microbial investigation. Agreements from the ethics committee of the “*Institut Fédératif de Recherche*, IFR48” and the “*Institut Hospitalo-Universitaire*, IHU-2017-007” were obtained to confirm the study procedure, as well as a written informed consent from parents of all patients^7^. All methods were performed in accordance with the relevant guidelines and regulations.

### *Clostridium* species isolation, *C*. *butyricum* qPCR detection

Stools were cultured for *Clostridium* species isolation using HS treatment based on the thermal resistance of spores^35^. Briefly, samples were suspended (v/v) in sterile phosphate-buffered saline by vortexing and heated for 20 minutes at 80 °C. Stools were then cultured on 5% Columbia sheep blood agar (Becton Dickinson®, USA) at 37 °C for 48 hours in an anaerobic atmosphere inside an anaerobic chamber. Provisional identification of all strains was performed by MALDI-TOF, then the amplification and sequencing of 16S rRNA gene was used to confirm this identification, as previously described^36^. Simultaneously, specific detection of *C*. *butyricum* qPCR was performed on DNA extracts obtained from stool samples of cases received after 2015, as described previously^7^. Data of qPCR and culture-based technique were analysed, the same 30 stool samples tested by culturomics method in Cassir *et al*. study being used as positive controls to validate the ability of spore-based method in the isolation of *C*. *butyricum*^7^.

### Core-genome and core-genome single-nucleotide polymorphism analysis

The genomic DNA of isolated *C*. *butyricum* was sequenced on MiSeq sequencer (Illumina, San Diego, CA, USA) using the paired-end strategy. Reads were assembled and scaffolded using SPAdes version 3.4.0^37^. Overall, 81 genomes were analysed, including 42 from strains isolated and sequenced in this study, 16 from previous isolates^7^, 5 strains isolated in our laboratory from humans as part of gut microbiota analysis^38^ and 18 from the Genbank database. Analysed genomes and their references are summarized in Supplementary Table S2. Protein sequences and nucleotide coordinates were predicted using the GeneMarkS platform (Genemark™, USA)^39^. Orthologous proteins analysis were performed using the ProteinOrtho software^40^. Core-genome coding DNA sequences were inferred from the pan-genome, concatenated and aligned using a Python script. SNPs were obtained from the core-genome using SNP-sites software^41^. Phylogenetic trees were generated using the maximum-likelihood method within PhyML^42^ and edited by Treegraph 2 software^43^. PHYLOViZ version 2.0 was used to generate a core-SNP eBURST phylogenetic tree based on expected numbers of nucleotide substitutions per variable site^44^. Scaled phylogenetic trees were procured using Adobe Photoshop CC 2018. BLASTP was retrieved to allow the identification of potential hemolysin sequences within the draft genomes of *C*. *butyricum*, their protein sequences are in Supplementary Material 1.

### Genotyping using multispacer sequence typing

MST was performed as described by Benamar *et al*.^10^. Briefly, DNA from patients’ isolates was extracted and amplified by standard PCR and then sequenced using 16 capillary sequencer 3130 XL (Applied Biosystems®, USA) and the BigDye Terminator v1.1 Cycle Sequencing kit (Applied Biosystems®, USA). For *C*. *butyricum* genomes available online, intergenic spacers were extracted by *in Silico* BLASTn.

### Statistical analysis

Statistical analysis was performed using the SPSS® statistics software 2016 (IBM, NY, USA). Mean and standard deviation were used to describe continuous variables. Percentage and number of events were used for quantitative variables. Student *t*-test or Mann-Whitney *U* test was used to perform two-group comparisons for quantitative variables. The chi-square (Mantel-Haenszel) test was used to perform two-group comparisons for qualitative variables.

## Supplementary information

Supplementary tables S1, S2, S3 and Supplementary figures S1, S2, S3, S4, S5, S6
