# Supplementary material for: Multidisciplinary evaluation of Clostridium butyricum clonality isolated from preterm neonates with necrotizing enterocolitis in South France between 2009 and 2017
Source: Sci Rep. 2019 Feb 14;9:2077. doi: 10.1038/s41598-019-38773-7 (PMC6376027; doi:10.1038/s41598-019-38773-7)
Supplement: Supplementary file 1 — Supplementary tables S1, S2, S3 and Supplementary figures S1, S2, S3, S4, S5, S6 [file 41598_2019_38773_MOESM1_ESM.docx]

Supplementary Informations

**Multidisciplinary evaluation of *Clostridium* *butyricum* clonality isolated from preterm neonates with necrotizing enterocolitis in South France between 2009 and 2017**

Michel Hosny^1^, Jacques Yaacoub Bou Khalil^1^, Aurelia Caputo^1^, Rita Abou Abdallah^2^, Anthony Levasseur^1^, Philippe Colson^1^, Nadim Cassir^1^ and Bernard La Scola^1^*.

# Supplementary Figures and Tables

**Table S1:** Relationship between the frequency of *Clostridium* *butyricum* and antibiotic administration in necrotizing enterocolitis and controls

|  | **Antibiotic administration** | |
| --- | --- | --- |
|  | Yes | No |
| **Necrotizing enterocolitis (n=88)** | | |
| Positive *C. butyricum* | 44 | 22 |
| Negative *C. butyricum* | 20 | 2 |
| *p value* | 0.027 | |
| **Controls (n=71)** | | |
| Positive *C. butyricum* | 3 | 5 |
| Negative *C. butyricum* | 26 | 37 |
| *p value* | 0.838 | |

**Table S2:** *Clostridium butyricum* genomes characteristics

| ***Clostridium butyricum* strains** | **Accession number/Reference** | **Origin** | **Location** | **Date of isolation** | **GC contents (%)** | **Contigs number** | **ORFs number** | **Genome length (base pair)** |
| --- | --- | --- | --- | --- | --- | --- | --- | --- |
| **NEC1** | ^1^ | Pn-NEC | NICU-1 | 2010 | 28.5 | 80 | 4,365 | 4,650,482 |
| **NEC2** | ^1^ | Pn-NEC | NICU-2 | 2010 | 28.5 | 80 | 4,372 | 4,648,403 |
| **NEC3** | ^1^ | Pn-NEC | NICU-1 | 2010 | 28.5 | 76 | 4,354 | 4,667,517 |
| **NEC4** | ^1^ | Pn-NEC | NICU-1 | 2010 | 28.5 | 82 | 4,414 | 4,666,341 |
| **NEC5** | ^1^ | Pn-NEC | NICU-2 | 2010 | 28.5 | 168 | 4,343 | 4,611,641 |
| **NEC7** | ^1^ | Pn-NEC | NICU-1 | 2011 | 28.5 | 168 | 4,338 | 4,599,670 |
| **NEC8** | GCA_001458815.1 | Pn-NEC | NICU-1 | 2010 | 28.5 | 26 | 5,509 | 4,742,905 |
| **NEC9** | ^1^ | Pn-NEC | NICU-1 | 2009 | 28.5 | 97 | 4,410 | 4,711,741 |
| **NEC10** | ^1^ | Pn-NEC | NICU-2 | 2009 | 28.5 | 51 | 4,098 | 4,498,679 |
| **NEC11** | ^1^ | Pn-NEC | NICU-4 | 2010 | 28.5 | 62 | 4,362 | 4,674,681 |
| **NEC12** | ^1^ | Pn-NEC | NICU-4 | 2010 | 28.5 | 77 | 4,380 | 4,690,695 |
| **NEC13** | ^1^ | Pn-NEC | NICU-3 | 2010 | 28.6 | 238 | 4,133 | 4,513,600 |
| **NEC14** | ^1^ | Pn-NEC | NICU-3 | 2011 | 28.5 | 36 | 4,080 | 4,501,358 |
| **NEC15** | ^1^ | Pn-NEC | NICU-3 | 2011 | 28.5 | 53 | 4,083 | 4,504,009 |
| **NEC16** | LS | Pn-NEC | NICU-1 | 2009 | 28.6 | 15 | 4,323 | 4,639,823 |
| **NEC17** | LS | Pn-NEC | NICU-1 | 2009 | 28.6 | 18 | 4,345 | 4,660,895 |
| **NEC18** | LS | Pn-NEC | NICU-1 | 2009 | 29 | 24 | 4,304 | 4,632,102 |
| **NEC19** | LS | Pn-NEC | NICU-1 | 2009 | 29 | 289 | 4,652 | 4,595,256 |
| **NEC20** | LS | Pn-NEC | NICU-1 | 2009 | 28.6 | 56 | 4,396 | 4,685,469 |
| **NEC21** | LS | Pn-NEC | NICU-2 | 2009 | 28.6 | 63 | 4,391 | 4,691,565 |
| **NEC23** | LS | Pn-NEC | NICU-3 | 2013 | 28.7 | 160 | 4,352 | 5,214,902 |
| **NEC29** | LS | Pn-NEC | NICU-1 | 2013 | 28.8 | 18 | 4,086 | 4,535,969 |
| **NEC35** | LS | Pn-NEC | NICU-1 | 2011 | 28.5 | 14 | 4,199 | 4,562,574 |
| **NEC39** | LS | Pn-NEC | NICU-2 | 2011 | 28.8 | 77 | 4,160 | 4,558,269 |
| **NEC45** | LS | Pn-NEC | NICU-1 | 2011 | 28.6 | 58 | 4,027 | 4,479,475 |
| **NEC46** | LS | Pn-NEC | NICU-1 | 2011 | 28.5 | 15 | 4,110 | 4,521,346 |
| **NEC47** | LS | Pn-NEC | NICU-1 | 2010 | 28.5 | 279 | 4,732 | 4,888,698 |
| **NEC48** | LS | Pn-NEC | NICU-1 | 2009 | 28.5 | 17 | 4,293 | 4,637,242 |
| **NEC49** | LS | Pn-NEC | NICU-1 | 2009 | 28.7 | 159 | 4,548 | 4,766,076 |
| **NEC50** | LS | Pn-NEC | NICU-1 | 2009 | 28.6 | 40 | 4,244 | 4,552,059 |
| **NEC52** | LS | Pn-NEC | NICU-1 | 2009 | 28.7 | 31 | 4,322 | 4,730,805 |
| **NEC57** | LS | Pn-NEC | NICU-1 | 2009 | 28.6 | 28 | 4,453 | 4,751,657 |
| **NEC58** | LS | Pn-NEC | NICU-2 | 2009 | 28.6 | 28 | 4,442 | 4,701,140 |
| **NEC59** | LS | Pn-NEC | NICU-1 | 2010 | 28.6 | 173 | 4,427 | 4,764,388 |
| **NEC61** | LS | Pn-NEC | NICU-1 | 2010 | 28.7 | 173 | 4,554 | 4,719,324 |
| **NEC62** | LS | Pn-NEC | NICU-1 | 2009 | 28.6 | 31 | 4,405 | 5,183,771 |
| **NEC64** | LS | Pn-NEC | NICU-1 | 2010 | 28.6 | 21 | 4,347 | 4,660,940 |
| **NEC65** | LS | Pn-NEC | NICU-1 | 2010 | 28.6 | 30 | 5,001 | 4,689,340 |
| **NEC66** | LS | Pn-NEC | NICU-1 | 2010 | 28.6 | 41 | 4,362 | 4,685,505 |
| **NEC70** | LS | Pn-NEC | NICU-1 | 2010 | 28.6 | 30 | 4,404 | 4,535,030 |
| **NEC71** | LS | Pn-NEC | NICU-1 | 2010 | 28.5 | 39 | 4,395 | 4,722,611 |
| **NEC72** | LS | Pn-NEC | NICU-1 | 2010 | 28.6 | 113 | 4,351 | 4,770,864 |
| **NEC73** | LS | Pn-NEC | NICU-1 | 2009 | 28.7 | 41 | 4,425 | 4,681,122 |
| **NEC74** | LS | Pn-NEC | NICU-1 | 2010 | 28.7 | 187 | 4,526 | 4,682,249 |
| **NEC75** | LS | Pn-NEC | NICU-1 | 2009 | 28.6 | 26 | 4,371 | 4,699,666 |
| **NEC76** | LS | Pn-NEC | NICU-1 | 2010 | 28.6 | 235 | 4,354 | 4,641,942 |
| **NEC78** | LS | Pn-NEC | NICU-1 | 2009 | 28.6 | 83 | 4,400 | 4,663,000 |
| **NEC82** | LS | Pn-NEC | NICU-1 | 2012 | 28.8 | 143 | 4,528 | 4,256,075 |
| **NEC83** | LS | Pn-NEC | NICU-1 | 2012 | 28.6 | 83 | 4,352 | 4,663,000 |
| **NEC84** | LS | Pn-NEC | NICU-1 | 2016 | 28.7 | 143 | 3,980 | 4,256,075 |
| **NEC88** | LS | Pn-NEC | NICU-1 | 2016 | 28.6 | 41 | 4,232 | 4,596,926 |
| **NEC89** | LS | Pn-NEC | NICU-1 | 2016 | 28.6 | 40 | 4,377 | 4,689,715 |
| **C13** | ^1^ | AS-Pn | NICU-3 | 2011 | 28.5 | 37 | 4,058 | 4,501,747 |
| **C14** | ^1^ | AS-Pn | NICU-3 | 2011 | 28.5 | 32 | 4,098 | 4,513,154 |
| **C 23** | LS | AS-Pn | NICU-1 | 2013 | 28.7 | 49 | 4,394 | 4,719,100 |
| **C 65** | LS | AS-Pn | NICU-1 | 2009 | 28.6 | 32 | 4,409 | 4,702,880 |
| **C 75** | LS | AS-Pn | NICU-1 | 2016 | 28.6 | 26 | 4,399 | 4,694,096 |
| **C 76** | LS | AS-Pn | NICU-1 | 2016 | 28.5 | 18 | 4,386 | 4,674,999 |
| **5521** | PRJNA20023 | Infant with botulism | Italy | NA | 28.8 | 123 | 4,230 | 4,540,699 |
| **60.E3** | PRJNA64855 | Asymptomatic adult | USA | 2013 | 28.8 | 10 | 4,255 | 4,644,398 |
| **AGR2140** | PRJNA185700 | Rumen | USA | 2015 | 28.5 | 39 | 4,183 | 4,550,822 |
| **BD04** | LS | Asymptomatic Bedouin | Saudi Arabia | 2013 | 28.5 | 39 | 4,101 | 4,462,170 |
| **CDC51208** | PRJNA301981 | NA | NA | NA | 28.7 | 3 | 4,243 | 4,639,914 |
| **CWBI1009** | PRJNA196989 | Anaerobic sludge | Belgium | 2013 | 28.5 | 340 | 4,151 | 4,491,619 |
| **DKU-01** | PRJNA193356 | Asymptomatic infant | Korea | 2013 | 28.6 | 79 | 4,151 | 4,519,722 |
| **DORA** | PRJNA221486 | AS-Pn | USA | 2013 | 28.6 | 264 | 3,782 | 4,014,159 |
| **DSM10702** | PRJNA196472 | Pig intestine | NA | 1880 | 28.5 | 207 | 4,372 | 4,596,811 |
| **DSM2477** | PRJEB9222 | Cotton wood tree | USA | 2011 | 28.5 | 62 | 4,123 | 4,516,699 |
| **DSM2478** | PRJEB9223 | Lake sediment | USA | 1993 | 28.5 | 61 | 4,227 | 4,627,604 |
| **E4str** | PRJNA34907 | Infant with botulism | Italy | 1984 | 28.7 | 13 | 4,396 | 4,758,422 |
| **HM-68** | PRJNA271049 | Chicken intestine | China | 2015 | 28.6 | 2 | 4,242 | 4,604,758 |
| **INCQS635** | PRJNA261798 | Soil | Brazil | 2013 | 28.5 | 231 | 6,093 | 4,407,025 |
| **JKY6D1** | PRJNA303063 | Pit mud | China | 2015 | 28.7 | 3 | 4,227 | 4,618,327 |
| **KW2** | LS | Children with Kwashiorkor | Senegal | 2015 | 28.5 | 170 | 4,219 | 4,512,068 |
| **KW10** | LS | Children with Kwashiorkor | Senegal | 2015 | 28.6 | 161 | 4,326 | 4,641,897 |
| **MAMOUTH** | LS | NA | NA | NA | 28.5 | 63 | 4,109 | 4,507,612 |
| **NOR33234** | GCA_000785185.1 | Elderly with diarrhoea | Japan | 2014 | 28.4 | 70 | 4,639 | 4,922,643 |
| **SU1** | PRJNA292365 | Rumen | India | 2014 | 28.6 | 434 | 6,040 | 4,879,486 |
| **TK520** | PRJNA327147 | Feces | China | 2014 | 28.7 | 3 | 4,047 | 4,463,546 |
| **Tn06** | LS | Asymptomatic adult | Saudi Arabia | 2013 | 28.5 | 70 | 4,341 | 4,674,236 |
| **TOA-1** | PRJNA314015 | Probiotics | India | 2012 | 28.7 | 3 | 4,187 | 4,597,202 |

GC: guanosine-cytosine; LS: Locally sequenced; ORF: open reading frame; bp: base-pair; Pn-NEC: Preterm neonates with NEC; AS-Pn: Asymptomatic preterm neonates.

**Table S3:** Advantage goals aimed by this study by comparison with that of Cassir *et al*., 2015 and Benamar *et al*., 2015

|  | Former analysis (reference) | Additional analysis (present study) |
| --- | --- | --- |
| Selected samples for qPCR | NEC: n=82  Controls: n=67 ^1^ | NEC: n=6  Controls: n=4 |
| Neonatal intensive care units | Marseille, Nice, Montpellier | Nîmes |
| Isolation of *C. butyricum* | n=16 ^1^ | n=42 |
| Isolation of other Clostridia | None ^1^ | n=25 |
| Sequenced genomes of *C.* *butyricum* | n=16 ^1,2^ | n=42 |
| Identified Clusters (techniques) | n=3 (MST, core-genome) ^2^ | n=2 (core-SNP) |

NEC: necrotizing enterocolitis, C: control.

**Figure S1:** *Clostridium butyricum* geographic relationship based on core-genome single-nucleotide polymorphism eBURST analysis


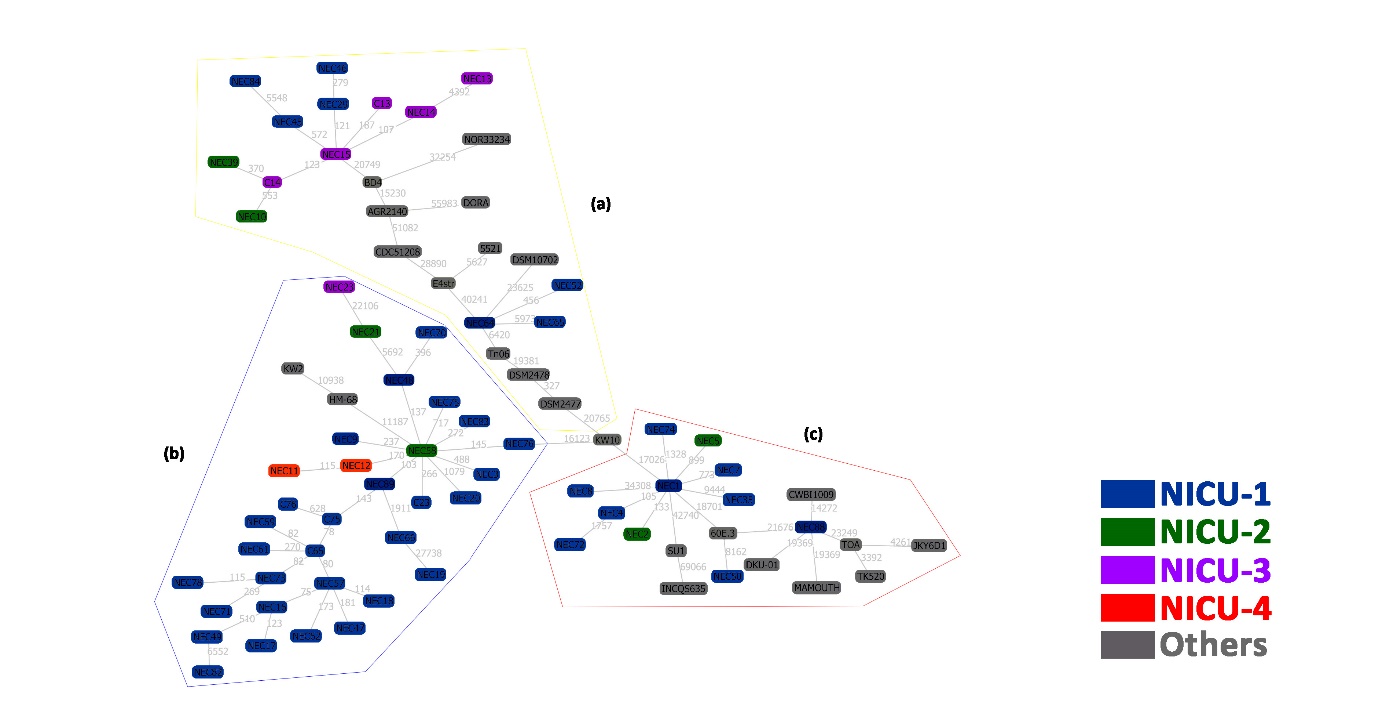


The color of strain names represents the geographic zone of isolation. (Marseille: NICU-1, NICU-2; Nice: NICU-3; Montpellier: NICU-4).

NEC: necrotizing enterocolitis, NICU: neonatal intensive care units.

**Figure S2:** *Clostridium butyricum* temporal relationship based on core-genome single-nucleotide polymorphism eBURST analysis


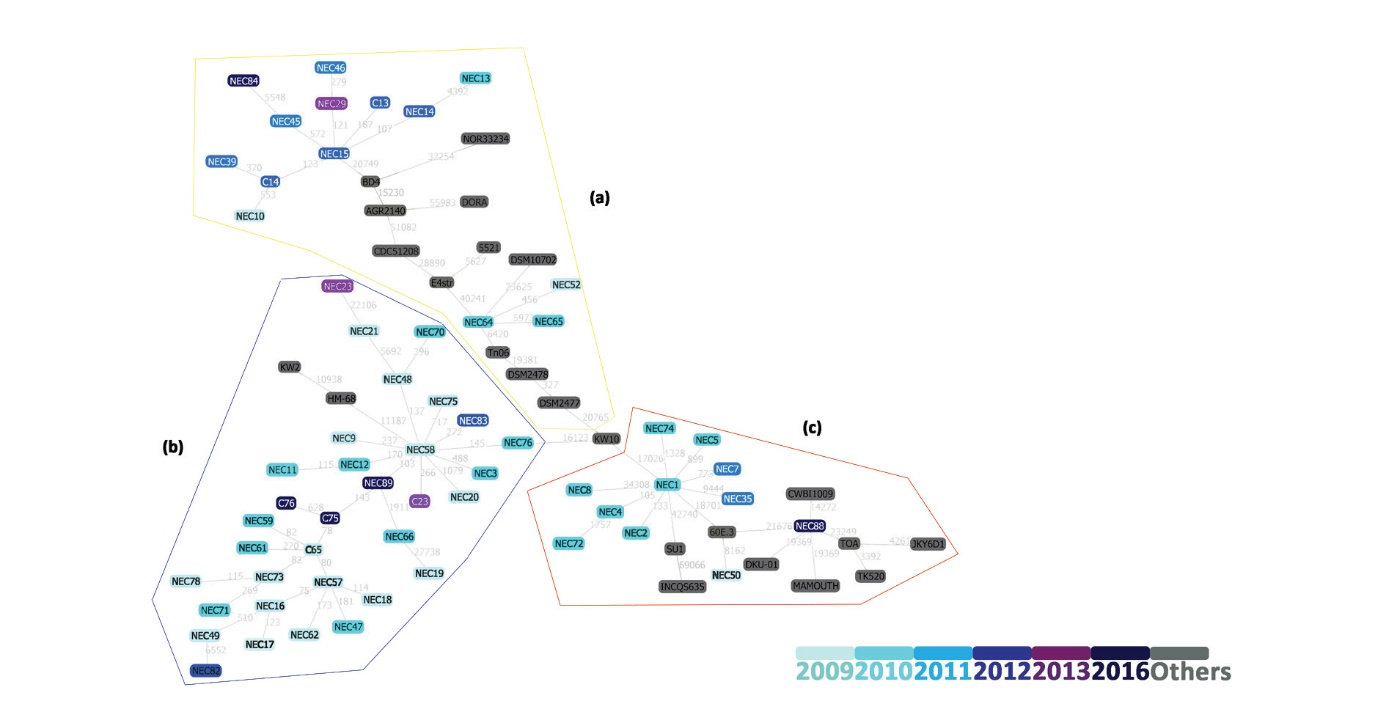


The color of strain names represents the timing of isolation.

NEC: necrotizing enterocolitis, C: control.

**Figure S3:** *Clostridium butyricum* relationship based on core-genome and core-genome single-nucleotide polymorphism phylogenetic analysis


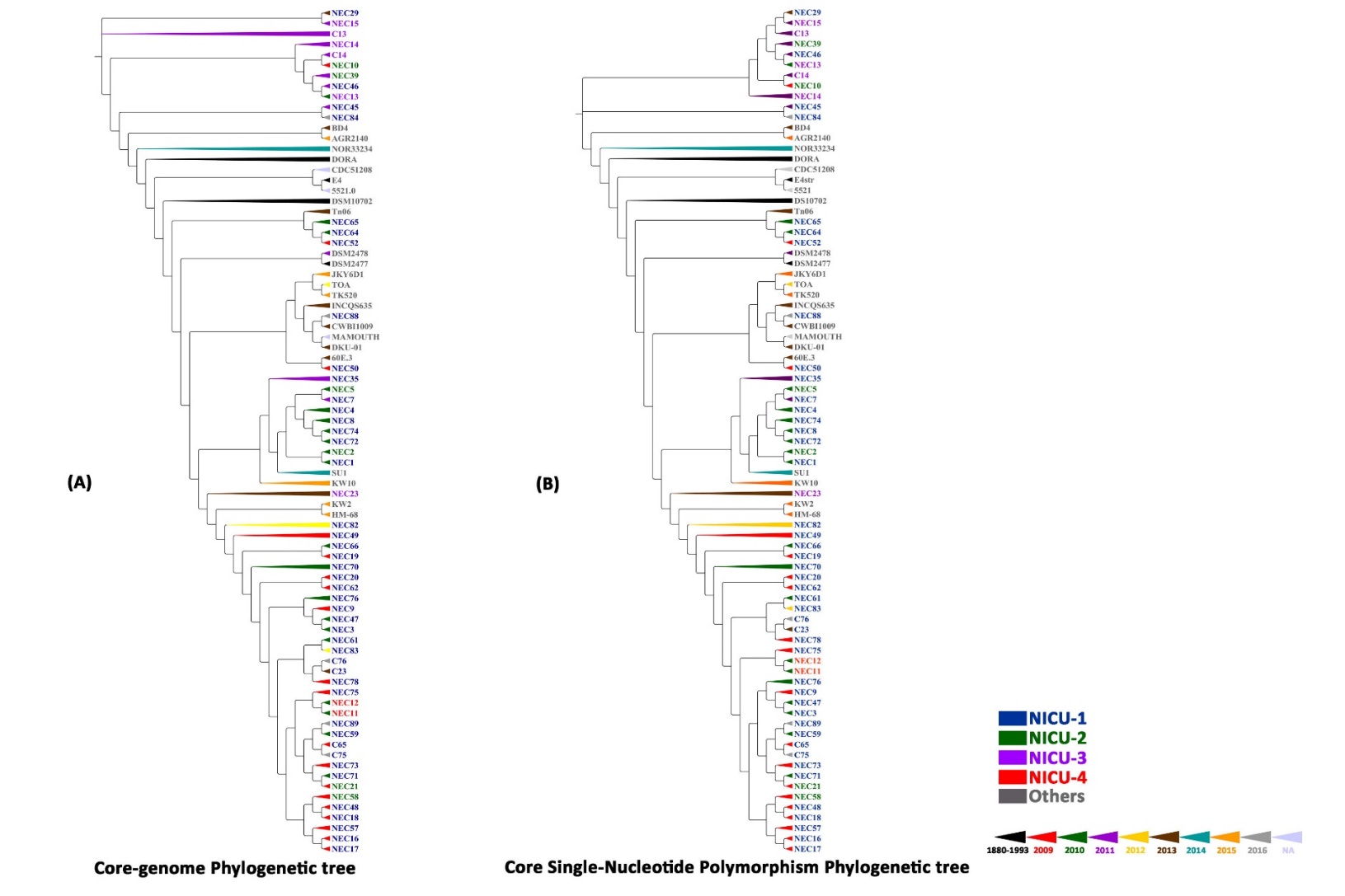


Maximum likelihood phylogenetic trees of NEC-associated and controls *Clostridium butyricum*. Branches nodes color represents the timing and the color of strain names represents geographic zone of strains isolation. (Marseille: NICU-1, NICU-2; Nice: NICU-3; Montpellier: NICU-4).

NEC: necrotizing enterocolitis, NICU: neonatal intensive care units.

**Figure S4:** *Clostridium butyricum* geographic relationship based on multispacer sequence typing eBURST analysis


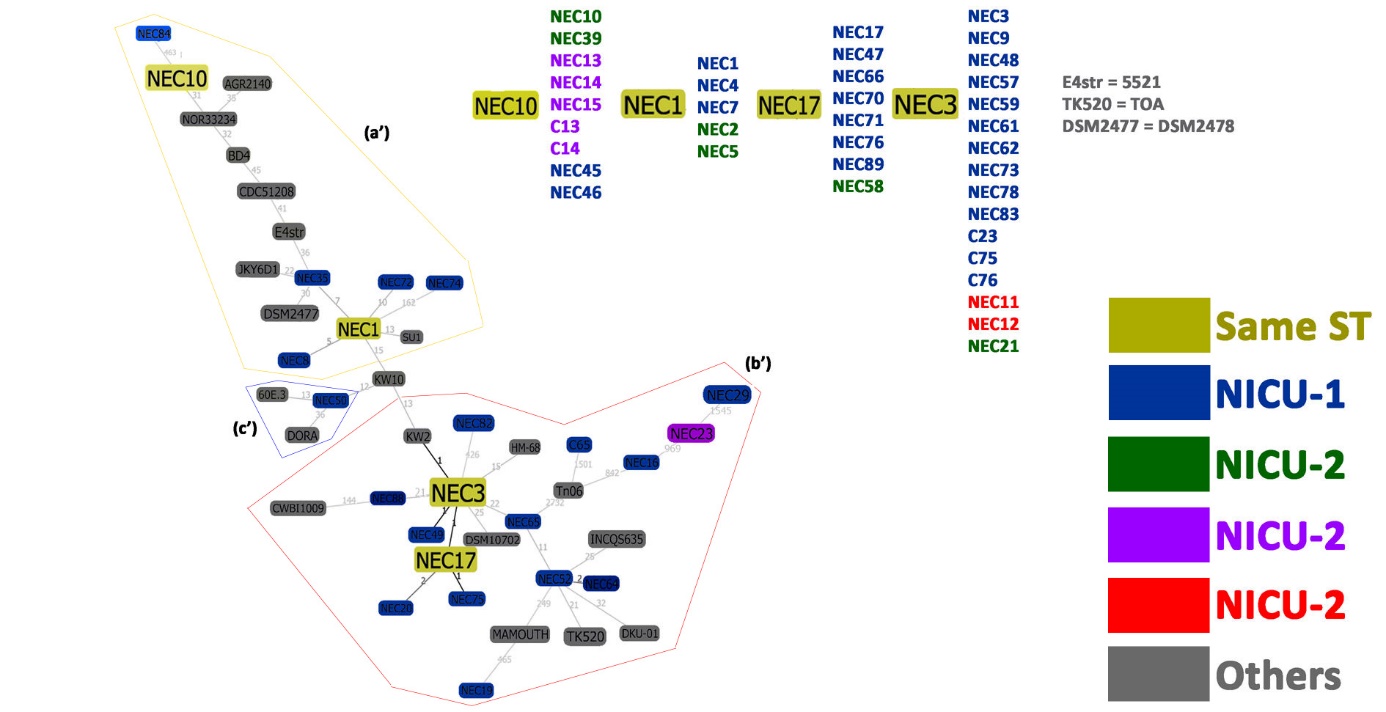


The color of strain names represents the geographic zone of isolation. (Marseille: NICU-1, NICU-2; Nice: NICU-3; Montpellier: NICU-4).

NEC: necrotizing enterocolitis, NICU: neonatal intensive care units.

**Figure S5:** *Clostridium butyricum* temporal relationship based on multispacer sequence typing eBURST analysis


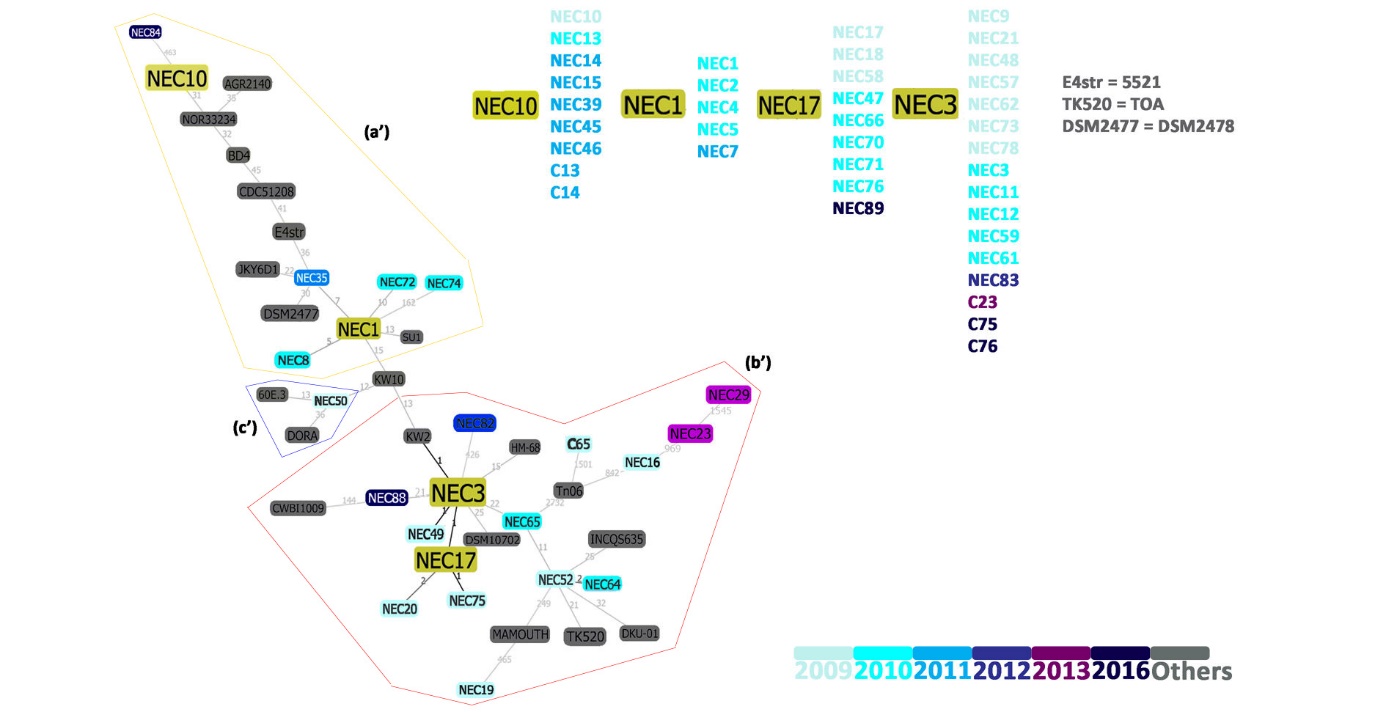


The color of strain names represents the timing of isolation.

NEC: necrotizing enterocolitis.

**Figure S6:** Distribution of necrotizing enterocolitis patients depending on the time and geographical origin


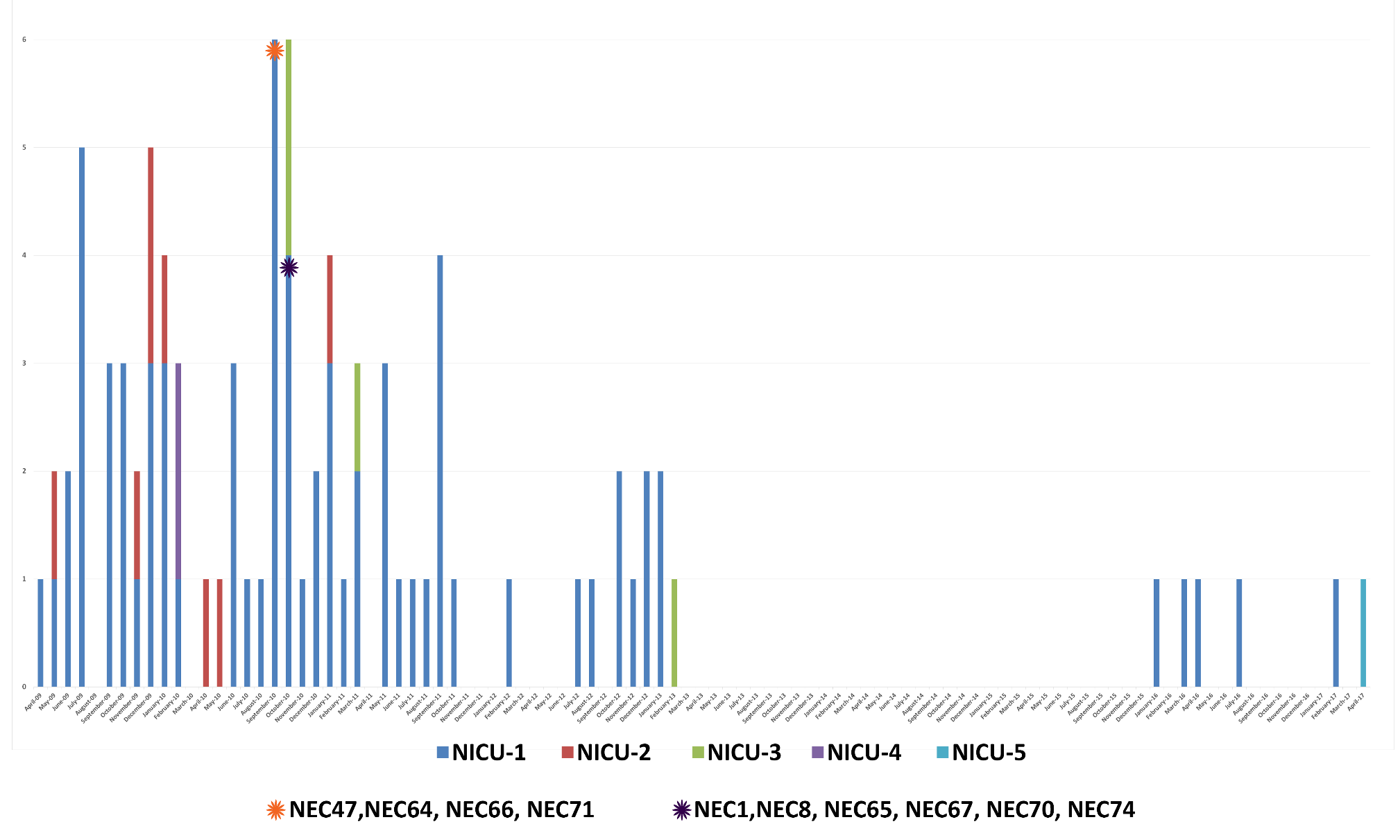


NEC: necrotizing enterocolitis, NICU: neonatal intensive care units.

**References**

1. Cassir, N. *et al.* *Clostridium* *butyricum* Strains and Dysbiosis Linked to Necrotizing Enterocolitis in Preterm Neonates. *Clin. Infect. Dis.* **61,** 1107–1115 (2015).

2. Benamar, S. *et al.* Multi-spacer typing as an effective method to distinguish the clonal lineage of *Clostridium* *butyricum* strains isolated from stool samples during a series of necrotizing enterocolitis cases. *J. Hosp. Infect.* **95,** 300–305 (2016).
